# Host response to cholestyramine can be mediated by the gut microbiota

**DOI:** 10.20517/mrr.2023.82

**Published:** 2024-07-11

**Authors:** Nolan K. Newman, Philip M. Monnier, Richard R. Rodrigues, Manoj Gurung, Stephany Vasquez-Perez, Kaito A. Hioki, Renee L. Greer, Kevin Brown, Andrey Morgun, Natalia Shulzhenko

**Affiliations:** ^1^Department of Pharmaceutical Sciences, College of Pharmacy, Oregon State University, Corvallis, OR 97331, USA.; ^2^Department of Biomedical Sciences, Carson College of Veterinary Medicine, Oregon State University, Corvallis, OR 97331, USA.

**Keywords:** Microbiome, *Lachnospiraceae*, *Muribaculaceae*, cholestyramine, network, hypercholesterolemia

## Abstract

**Background:** The gut microbiota has been implicated as a major factor contributing to metabolic diseases and the response to drugs used for the treatment of such diseases. In this study, we tested the effect of cholestyramine, a bile acid sequestrant that reduces blood cholesterol, on the murine gut microbiota and metabolism. We also explored the hypothesis that some effects of this drug on systemic metabolism can be attributed to alterations in the gut microbiota.

**Methods:** We used a Western diet (WD) for 8 weeks to induce metabolic disease in mice, then treated some mice with cholestyramine added to WD. Metabolic phenotyping, gene expression in liver and ileum, and microbiota 16S rRNA genes were analyzed. Then, transkingdom network analysis was used to find candidate microbes for the cholestyramine effect.

**Results:** We observed that cholestyramine decreased glucose and epididymal fat levels and detected dysregulation of genes known to be regulated by cholestyramine in the liver and ileum. Analysis of gut microbiota showed increased alpha diversity in cholestyramine-treated mice, with fourteen taxa showing restoration of relative abundance to levels resembling those in mice fed a control diet. Using transkingdom network analysis, we inferred two amplicon sequence variants (ASVs), one from the *Lachnospiraceae* family (ASV49) and the other from the *Muribaculaceae* family (ASV1), as potential regulators of cholestyramine effects. ASV49 was also negatively linked with glucose levels, further indicating its beneficial role.

**Conclusion:** Our results indicate that the gut microbiota has a role in the beneficial effects of cholestyramine and suggest specific microbes as targets of future investigations.

## INTRODUCTION

Hypercholesterolemia (excessive amounts of cholesterol in the blood) is present in more than 25% of American adults over the age of 20, reaching almost 40% incidence in certain sex, race, and age groups^[1]^. Individuals with familial hypercholesterolemia are at a higher risk of developing cardiovascular diseases^[2]^ and 60%-70% of patients with type 2 diabetes mellitus (T2D) also have dyslipidemia^[3]^. An increase in foods rich in animal fats and sugars, termed a “Western diet” (WD), has led to an increasing number of people with hypercholesterolemia and T2D, making it increasingly important to understand how anti-hypercholesterolemic drugs act in the body. Recently, it has become clear that both antidiabetics and anti-hypercholesterolemic drugs cause alterations in the host microbiota. For example, atorvastatin, a commonly used HMG-CoA (β-Hydroxy β-methylglutaryl-CoA) reductase inhibitor, has been shown to alter the gut microbiota of rats fed a high-fat diet^[4]^.

Cholestyramine, another anti-hypercholesterolemic drug, acts by binding to bile acids in the gut and preventing their absorption, increasing the excretion of bile acids and fat in the stool. This, in turn, increases cholesterol metabolism in the liver, thus reducing blood cholesterol levels^[5]^. Interestingly, cholestyramine has also been shown to improve serum glucose levels in rats^[6]^, which is a common marker of diabetes.

Current research demonstrates that the gut microbiota has an important role in various aspects of the host metabolism, including regulation of intestinal metabolic gene expression^[7]^, blood glucose and glucose intolerance^[8]^.

A study published in 1975^[9]^ showed a decrease in anaerobic bacteria after one week of cholestyramine, but comprehensive analysis of microbiota was not possible back then. More recently, it was reported that incubation of gut content with cholestyramine *ex vivo* resulted in an increased abundance of the pathogen *Clostridium difficile*^[10]^. Due to the interrelationships between bile acids and the gut microbiota^[11]^, it has been hypothesized that cholestyramine’s bile acid sequestration mechanism would cause changes in the gut microbiota composition^[12]^. We sought to determine whether cholestyramine would alter the composition of the gut microbiota in mice fed a high animal fat, high sugar diet (WD). Our results demonstrate that in WD-fed animals, cholestyramine treatment results in the amelioration of metabolic parameters, gene expression in the liver and gut, and large shifts in the gut microbiota composition, increasing microbiota diversity. Furthermore, we applied a computational approach, Transkingdom Network Analysis (TkNA), that we previously developed and successfully used^[13-16]^ to infer specific bacteria that can be critical for the effect of cholestyramine. This analysis identified microbial species from families *Lachnospiraceae* and *Muribaculaceae* that may play a role in facilitating the response to cholestyramine. Altogether, these results suggest that cholestyramine may at least partially act by altering the gut microbiota and its mechanism may be dependent on the relative abundance of certain bacterial species.

## METHODS

### Mice and cholestyramine treatment

**Figure 1:**
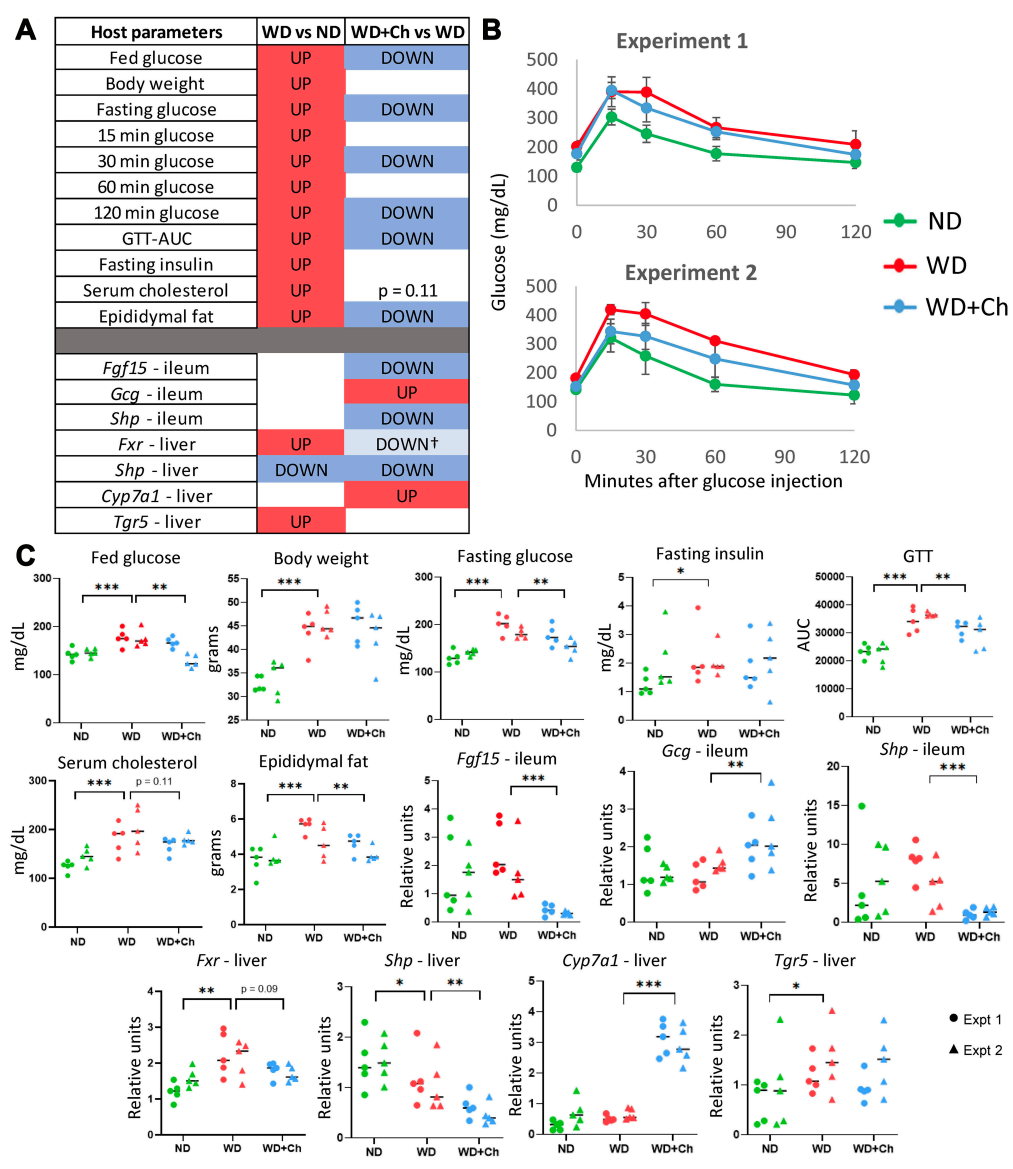
Metabolic and gene expression changes induced by WD compared to a ND and WD+Ch compared to only WD in mice. Mice were separated into three experimental groups across two experiments (*n* = 5 per group, per experiment). Statistics were performed on the pooled samples from both experiments, but the fold change direction for each parameter was required to be consistent across experiments. Data for each experiment are shown separately per treatment group. (A) Summary table of significantly changed host parameters (phenotypes: one-tailed Mann-Whitney U *P*-value < 0.05) and genes (two-tailed Mann-Whitney U *P*-value < 0.05) that are consistent in fold change direction across both experiments; (B) GTT plots demonstrating the changes in blood glucose levels over the first 2 h after glucose injection; (C) dot plots of phenotypes and gene expression that demonstrated significant differences in fold changes. Circles represent experiment 1 and triangles represent experiment 2. *P < 0.05; **P < 0.01; ***P < 0.001; †*P* < 0.1. WD: Western diet; ND: normal diet; WD+Ch: WD with cholestyramine; GTT: glucose tolerance test.

Work on mice was carried out in accordance with the Guide for the Care and Use of Laboratory Animals as adopted by the U.S. National Institutes of Health, and was approved by Oregon State University’s Animal Care and Use Committee (protocol IACUC-2021-0202). Seven-week-old, Specific Pathogen-Free (SPF), C57BL/6J male mice were purchased from Jackson Laboratory (Bar Harbor, Maine) in separate batches of 15 mice six months apart and housed in the controlled environment (12 h daylight cycle) of the Laboratory Animal Resources Center (LARC) at Oregon State University with ad libitum access to food and water. After one week of acclimatization, cages of 5 mice per group were randomly assigned to either a WD D12451 containing 40 kcal% lard and 17 kcal% sucrose or a matching normal diet (ND) D12450K produced by Research Diets (New Brunswick, NJ). After 8 weeks, one group of mice was switched from WD alone to the same WD supplemented with 2% cholestyramine (20g/kg of diet, Sigma)^[17]^ for 10-11 weeks [Supplementary Figure 1]. This dose roughly corresponds to the prescribed human dose, considering the accepted dose conversion between humans and mice^[18]^. Based on our previous studies^[8]^ and to ensure reproducibility, two independent experiments (*n* = 5 mice per group per experiment) were performed six months apart. In experiment 2, mice were supplemented with cholestyramine for 11 weeks instead of 10 due to personal health issues, but the results showed high concordance [Figure 1]. Two independent experiments were used to help ensure the reproducibility of findings (see Section “Network reconstruction and identification of key nodes”). No mice were excluded and no blinding was used, but different people did experiments and analyzed data. Body weight and feed intake were measured each week. Following the conclusion of the study, mice were euthanized using a method accepted in the Guide for the Care and Use of Laboratory Animals (cervical dislocation followed by pneumothorax) and tissues were collected. None of the mice needed euthanasia prior to the planned end of studies, but criteria were established for this, which were pain or distress levels requiring euthanasia as judged by the attending veterinarian.

### Glucose tolerance tests

Mice were fasted for 6 h during the light phase with free access to water. A concentration of 2 mg/kg body weight glucose (Sigma-Aldrich) was injected intraperitoneally. Blood glucose was measured at 0 (immediately before glucose injection), 15, 30, 60, and 120 min with a Freestyle Lite glucometer (Abbot Diabetes Care).

### Serum fasting insulin

Mice were fasted for 6 h with free access to water. Fasting blood was collected via submandibular bleed and serum was separated after 30 min clotting and 3,000 rpm centrifugation for 15 min. Insulin in serum was measured with Mouse Insulin ELISA Kit (Crystal Chem, cat# 90080) according to manufacturer’s protocol.

### Serum cholesterol

Serum cholesterol was measured using a Cholesterol Assay Kit (Thermofisher, cat# EEA026) as per the manufacturer’s instructions. Non-fasted serum was used.

### Bacterial DNA extraction and 16S rRNA gene library preparation

For microbial measurements, fresh stool pellets were collected and immediately stored at -80 °C. To extract microbial DNA, frozen fecal pellets (2-3 per mouse) were resuspended in 1.4 mL ASL buffer (Qiagen) and homogenized using an OMNI Bead Ruptor (OMNI International) first with 7-8 ceramic 2.8 mm beads (speed 5.65 m/s, 30 s twice) followed by 0.2 mL of 0.5 mm glass beads (speed 3.25 m/s, 5 min). Next, DNA was extracted from the entire resulting suspension using a QiaAmp Mini stool kit (Qiagen, cat# 51504) according to the manufacturer’s protocol. DNA was quantified using the Qubit broad range DNA assay (Thermofisher, cat# Q32850). The V4 region of the 16S rRNA gene was amplified using a standard protocol^[19]^ with bacterial universal primers (515F: GTGCCAGCMGCCGCGGTAA; 806R: GGACTACHVGGGTWTCTAAT) in 25-cycle PCR reactions with negative control showing no amplification as in our previous work^[20]^. Individual samples were barcoded, pooled to construct the sequencing library, and then sequenced at the Center for Genome Research (Oregon State University) using an Illumina Miseq Instrument (Illumina, San Diego, CA) to generate paired-end 250 bp reads.

### Tissue collection, RNA preparation and gene expression analysis

Mouse liver and ileum samples (2-3 cm) were collected at the end of the study, snap-frozen and stored at -80 °C. For RNA isolation, ilea were homogenized in RLT buffer using the OMNItip tissue disruptor (OMNI International, cat# 321250). RNA was then extracted using the QiaCube RNeasy kit with Qiashredder as per the manufacturer’s instructions. Mouse liver samples were homogenized in Trizol using the OMNItip tissue disruptor, and 200 uL of chloroform was added to each sample. Each tube was then centrifuged at 4 °C and 12,000 *g* for 15 min. RNA was then extracted from the upper aqueous layer using the QiaCube RNeasy kit with Qiashredder (Qiagen, cat# 79656), following the same protocol as for the ileum samples. On-column DNA digestion was used for both types of tissues. Concentrations of RNA were measured using the Quant-IT RNA BR Assay Kit (Life Technologies). Reverse transcription of 500 ng RNA into cDNA was performed using the qScript cDNA Synthesis Kit (Quanta Biosciences) following the manufacturer’s protocol. Primer master mixes were prepared for Quantitative Real-Time PCR with the PerfeCTa SYBR Green PCR Kit (QuantaBio) with Polr2c as a housekeeping gene. Primers used for each gene are included in Supplementary Figure 2. qRT-PCR was performed on the StepOnePlus Real-Time PCR system (Applied Biosystems) with the following protocol: samples were heated to 95 °C and held for 30 s, followed by 40 cycles of 95 °C for 3 s and 60 °C for 20 s. Gene expression values of each gene were calculated using the standard curve method^[21,22]^. For this, gene expression standards for ileum and liver samples were created for each tissue by pooling cDNA from all 30 mice included in two experiments. A 1:10 dilution of the standard was created to plot the standard curve and 2^-Ct^ values of samples were used to calculate cDNA concentrations for each gene. Next, gene expression of target genes was calculated relative to the housekeeping gene (Polr2c) for each sample.

### 16S rRNA gene (amplicon) sequencing analysis

Data of 16S rRNA gene (amplicon) sequencing for both experiments were uploaded to SRA (Sequence Read Archive) and can be found through the Bioproject accession number PRJNA686421. The QIIME 2^[23]^ bioinformatics pipeline (v. 2018.8.0) was used to demultiplex and quality filter the forward-end fastq files. Denoising was performed using DADA2^[24]^. The mean sequencing depth per sample after filtering steps was 51,307.4 and SD+/- 11,596.9. Microbes with a frequency above 99.95% of cumulative abundance across all samples in an experiment were included in further analysis. Common amplicon sequence variants (ASVs) within this threshold in both experiments were relativized per million and then quantile normalized.

Taxonomy was assigned to theASVs using multiple methods. Initially, the pre-trained naïve Bayes taxonomy classifier was used against the Greengenes 13_8 99% reference sequences^[25]^ for all ASVs.

Further analysis aimed at better resolution of classification of the top two candidates (ASV1 and ASV49) was performed using the pre-trained naïve Bayes taxonomy classifier against the Silva 138 99% reference sequences and alignment of sequences via BLAST using the classify-consensus-blast step in QIIME 2. The sequences were aligned using the provided silva-138-99-seqs.qza reference database and silva-138-99-tax.qza files supplied through QIIME 2. Furthermore, classification was also performed through the SILVA ACT online tool (https://www.arb-silva.de/), as well as through the online BLAST tool^[26]^. All taxonomic classification results of ASV1 and ASV49 are reported in Supplementary Table 1.

For beta diversity analysis, the quantile normalized ASV tables were uploaded to MicrobiomeAnalyst^[27,28]^. PCoA plots were generated using the Bray-Curtis dissimilarity distance method at the feature level and the analysis of similarities (ANOSIM) was calculated between treatment groups. Alpha diversity was measured using the Shannon diversity index and a Mann-Whitney U test was performed to compare the WD+Ch and WD groups. Bacterial families that were significantly changed between the WD and ND groups, as well as WD+Ch and WD groups, were determined using a two-tailed Mann-Whitney U test (*P* < 0.05). Results of those families that had consistent fold change between the two experiments were analyzed using Fisher’s combined probability test as in our previous work^[8]^.

Prior to creating the heatmaps, raw ASVs were relativized per sample, and then mean normalized across all samples. Following this, the data were filtered to contain only microbes that belonged to one of three categories: (1) The ASV significantly (two-tailed Mann-Whitney U test *P*-value < 0.1) increased (or decreased) in the WD group compared to the ND group, and significantly changed in the opposite direction in the WD+Ch groups compared to the WD group; (2) The ASV significantly increased (or decreased) in the WD+Ch group compared to ND and WD, but there was no difference when comparing the ND and WD group to one another; (3) The ASV significantly increased (or decreased) in the WD and WD+Ch group compared to the ND group, but there was no difference when comparing the WD and WD+Ch group to one another. The ASV was required to behave similarly across both experiments for inclusion in the heatmap. After creating these three groups, heatmaps for experiment 1 were generated using the online Morpheus heatmap generator (https://software.broadinstitute.org/morpheus). Hierarchical clustering using the Euclidean distance metric and complete linkage was performed on rows and each row was scaled based on the maximum and minimum values of the row. Heatmaps for experiment 2 reflect the same row order as in experiment 1, meaning clustering was not performed on experiment 2.

### Network reconstruction and identification of key nodes

Differentially expressed genes and significant metabolic parameters were found by first checking for the same fold change direction in both experiments, then median normalizing each parameter per experiment and pooling. Host parameters were considered to be significant if the FDR-corrected one-tailed Mann-Whitney U p-value was less than 0.05 between the WD+Ch and WD groups. Significant ASVs were found similarly, although without median normalizing of the quantile normalized data prior to pooling and using a two-tailed Mann-Whitney U FDR threshold of 0.1. In the WD+Ch group, Spearman correlations were then performed between each metabolic parameter, gene, and ASV. Correlations were filtered based on the following criteria: (1) same direction of correlation (+/-) across both experiments prior to pooling; (2) Spearman correlation *P*-value < 0.05, which corresponded to a Benjamini-Hochberg FDR of 0.25; and (3) no correlation inequalities^[29,30]^. Networks were visualized using Cytoscape v3.7.2^[31]^. The Python module NetworkX v2.2^[32]^ was used to calculate bipartite betweenness centrality (BiBC) and degree between groups, as well as to randomly generate the 10,000 networks used in validating the BiBC and degree results. BiBC was calculated as previously described^[33,34]^.

Random networks were generated as in previous publications^[35,36]^. In short, binomial random (Erdos-Renyi) networks were generated from the G (m, n) ensemble with m = 154 (to match the number of edges in the real network) and n = 84 (the number of nodes). No self- or multi-edges were allowed. The online tool Plotly (https://plot.ly/) was used to plot the 2D contour histogram of the BiBC-degree distribution. Probability density was used as a measurement of the likelihood of randomly finding a node (specifically ASV49) with the given BiBC and degree (or higher). A large value (i.e., a dark-colored space in the contour map) indicates that a node in that area typically occurs in random networks size-matched to the network generated from the data.

## RESULTS

### Cholestyramine restores systemic parameters associated with metabolic disease

To study the effect of cholestyramine on the host, eight-week-old male C57BL/6J mice were first acclimated to their respective assigned diets (see methods) for 8 weeks. The study was then continued for 10-11 weeks, where mice assigned to the ND or WD groups did not receive Cholestyramine treatment while those assigned to the WD+Chol group were supplemented with 2% cholestyramine [Supplementary Figure 1]. Systemic parameters associated with T2D were measured. The control groups were either fed a WD or ND for 18-19 weeks. Mice on a WD demonstrated an increase in 11 parameters associated with metabolic disease seen in diabetic individuals, compared to the ND group [Figure 1 and Supplementary Table 2]. Eight of these parameters decreased, six of which were significant (one-sided Mann-Whitney U FDR < 0.1), in the WD+Ch group, compared to the WD group. In particular, parameters associated with glucose metabolism (i.e. fed glucose levels, fasting glucose levels, and glucose levels at various time points after injecting glucose; Figure 1A and B, Supplementary Figure 3) were decreased by cholestyramine (WD+Ch), along with epididymal fat mass. Although not statistically significant because of high variability, serum cholesterol levels dropped as expected with cholestyramine treatment (*P*-value = 0.11).

### Cholestyramine changes the expression of genes involved in bile acid metabolism

Due to cholestyramine’s mechanism of action (sequestration of bile acids and limiting reabsorption), we hypothesized that treatment with cholestyramine would cause a change in the expression of genes involved in the classical bile acid synthesis pathway and bile acid metabolism^[37,38]^. The group of mice on a WD exhibited increased expression of farnesoid X receptor [*Fxr*, also known as nuclear receptor subfamily 1 group H member 4 (*Nr1h4*)] and *Tgr5* (Takeda G protein-coupled receptor 5, also known as *Gpbar1*, G protein-coupled bile acid receptor 1), and decreased expression of small heterodimer protein (*Shp*) (also known as *Nr0b2*, nuclear receptor subfamily 0 group B member 2) in the liver [Figure 1A and C]. Of the genes measured in the classical bile acid metabolism pathway, three in the ileum were altered by treatment [fibroblast growth factor 15 (*Fgf15*), glucagon (*Gcg*), and *Shp*]. *Fgf15* and *Shp* in the ileum decreased expression with treatment, while *Gcg* in the ileum increased [Figure 1C]. Only *Shp* and cytochrome P450 family 7 subfamily A member 1 (*Cyp7a1*) were significantly altered in the liver after treatment, which exhibited decreased and increased expression, respectively. Gene expression of *Fxr* in the liver showed a trend toward decreased expression (*P*-value = 0.09).

### Cholestyramine alters the gut microbiota composition

**Figure 2:**
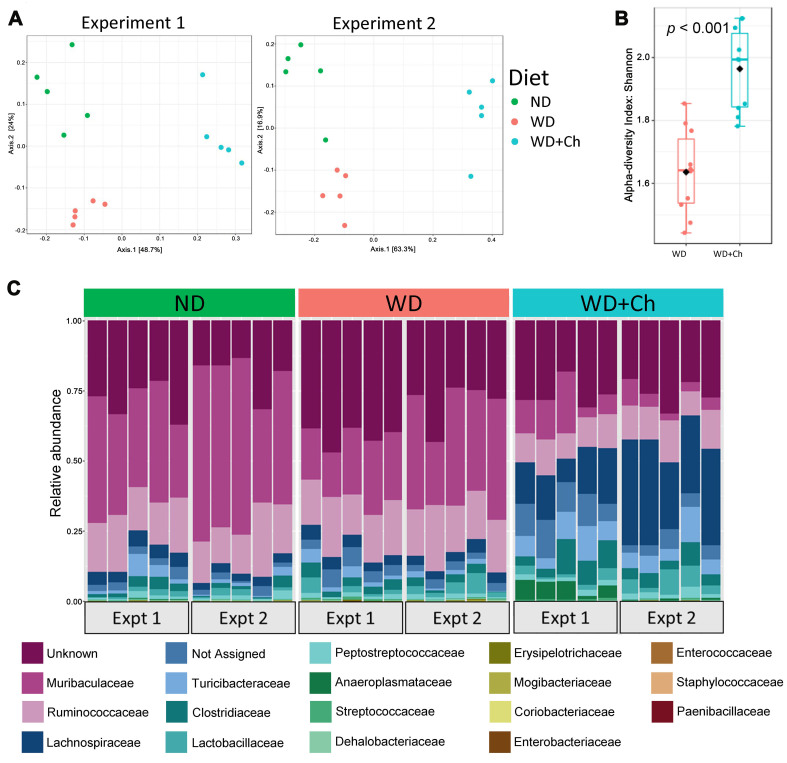
Effect of Ch on microbiota in WD fed mice: (A) PCoA plots demonstrating there is a shift in microbial composition in WD-fed mice administered cholestyramine (WD+Ch, blue) from that of the WD-fed, cholestyramine-free group (red) and mice administered a normal chow diet (green). ANOSIM R-values for Experiment 1 and Experiment 2 are 0.957 and 0.966, respectively, and each has a *P*-value < 0.001; right plot shows alpha diversity Shannon index; (B) family level alpha diversity is significantly higher in the WD+Ch group (Mann-Whitney U *P*-value < 0.001); (C) relative abundances of the families found in each mouse. Ch: Cholestyramine; WD: Western diet; WD+Ch: WD with cholestyramine.

We next asked whether changes in the gut microbiota could be in part responsible for the restoration of metabolic parameters observed following treatment with cholestyramine. We thus performed an analysis of the microbial 16S rRNA genes in the stool samples of all mice in both experiments. Microbiomes of the cholestyramine-treated mice separated from those in the ND and WD samples primarily on PC1, which accounted for 48.7% and 63.3% of the variability seen in experiments 1 and 2, respectively [Figure 2A]. All groups demonstrated a significant difference in microbial composition in both experiment 1 (ANOSIM R-value 0.957, *P*-value < 0.001) and experiment 2 (ANOSIM R-value 0.966, *P*-value < 0.001). The ND and WD samples separated mainly on PC2, which accounted for 24% and 16.9% of the differences seen in experiments 1 and 2, respectively. All groups clustered distinctly and there was no overlap between groups. Comparisons between WD and ND groups, as well as WD+Ch and WD groups, can be seen in Supplementary Figure 4A. Alpha diversity increased significantly in the WD+Ch group compared to the WD group [Figure 2B]. Concordantly, the number of observed ASVs was significantly lower in the WD group compared to the other two groups [Supplementary Figure 4B].

Family level relative abundances [Figure 2C] showed that only three families were consistently changed by WD across both experiments and none of them were consistently reversed back towards levels of ND mice by cholestyramine [Supplementary Table 3].

**Figure 3:**
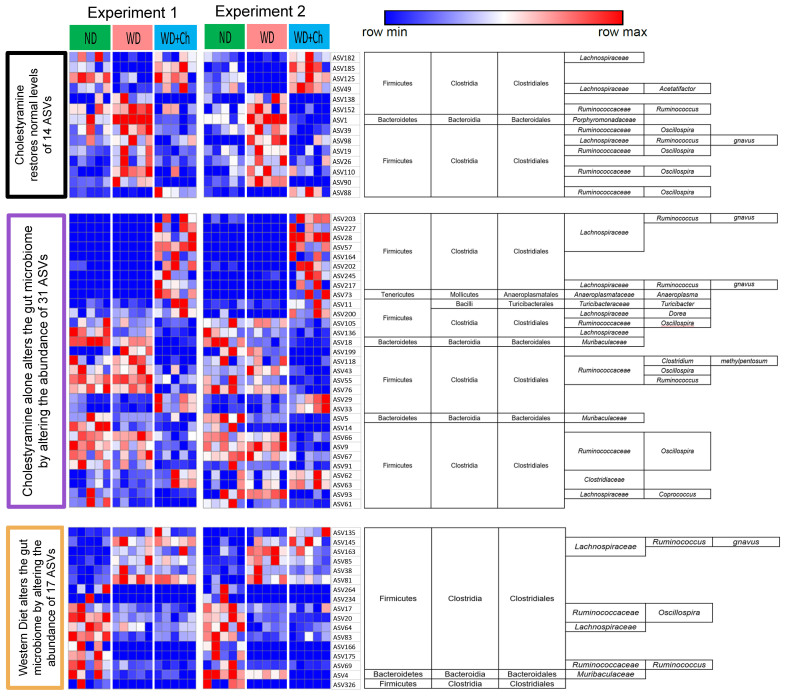
Heatmap of relative abundances of ASVs significantly changed by Ch and the WD which can be assigned into three different groups based on the changes in relative abundance between the groups. Group 1 (black): 14 ASV abundances are significantly reversed back toward ND levels after the introduction of cholestyramine to a WD. Group 2 (purple): abundance of 31 ASVs is altered by cholestyramine alone. Group 3 (orange):17 ASVs are altered by a WD, regardless of treatment with cholestyramine. Rows (ASVs) of heatmaps were clustered first by abundance in experiment 1 and the same order was used for experiment 2. Significant ASVs were required to be consistent in fold-change direction across both experiments (Mann-Whitney U *P*-value < 0.05). ASVs: Amplicon sequence variants; Ch: cholestyramine; ND: normal diet; WD: Western diet.

We next asked which of the ASVs could possibly contribute to the observed restoration of the six metabolic parameters, and classified ASVs into three distinct groups [Figure 3]. Group 1 refers to those ASVs that demonstrated opposite fold change direction between WD and WD+Ch treatments, meaning those that were increased (or decreased) by WD and decreased (or increased) in the WD+Ch group. Thus, the relative abundances of these ASVs were reversed toward normal levels by treatment with cholestyramine. Among the 14 ASVs found, 13 belonged to the order Clostridiales while one belonged to Bacteroidales. Group 2 contained those ASVs that were changed in only the WD+Ch treatment group, indicating these changes were independent of diet. Of the 31 ASVs, the majority were also Clostridiales. Finally, group 3 contained 17 ASVs that changed in the same direction in both the WD and WD+Ch compared to the ND group. The fact that these were not dependent on cholestyramine indicates they are potentially non-causal microbes for the response to cholestyramine.

Although we identified 14 ASVs that are restored by cholestyramine treatment, those that regulate the host parameters cannot be distinguished only by changes in their relative abundances. Thus, to identify potential causal microbes, we turned to reconstruct a regulatory network between the phenotypes, genes, and ASVs in the WD+Ch treated group.

### Transkingdom network analysis identifies two ASVs as potential regulators of the response to cholestyramine

**Figure 4:**
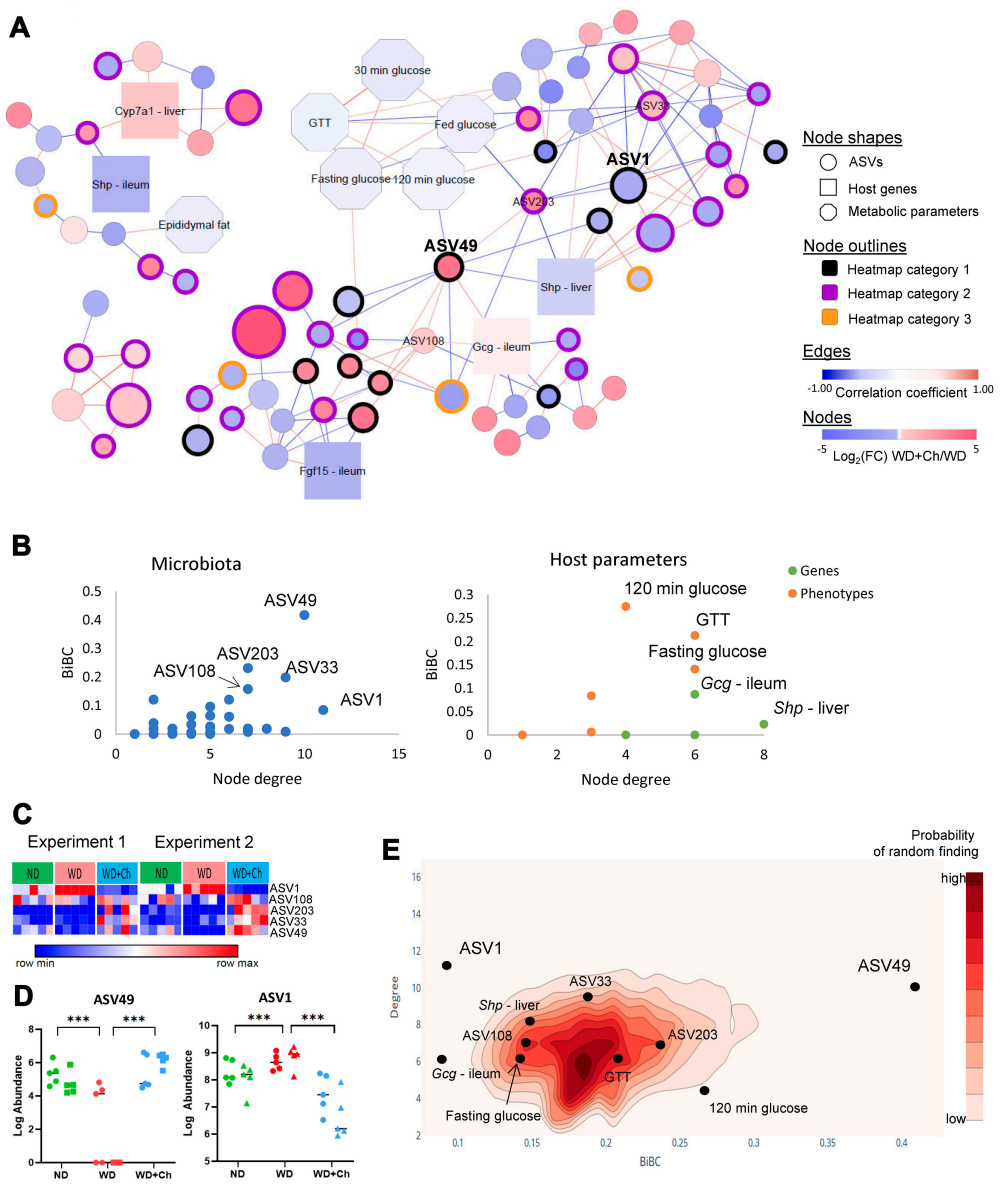
Transkingdom network analysis points to potential bacterial regulators of cholestyramine effects on the host. (A) Transkingdom network between genes, phenotypes, and ASVs. ASV outlines reflect the heatmap group from Figure 3 to which the microbes belong (heatmap #1 = black, #2 = purple, #3 = orange). Edges represent significant Spearman correlations (Spearman *P*-value < 0.05) and are colored based on Spearman’s rho coefficients (blue: negative correlation, red: positive correlation). Nodes are colored based on the fold-change direction (red: up, blue: down) of the node between the WD+Ch group and the WD group (Mann-Whitney U *P*-value < 0.05). ASV node size is based on log-transformed abundance; (B) BiBC-degree distribution of all nodes, the highest of which are labeled; (C) Heatmap of abundances of top five ASVs ranked on BiBC-degree distribution; (D) Abundances of the top two ASVs, ***P < 0.001; (E) 2D-contour histogram of the nodes with the highest BiBC and degree from 10,000 randomly generated networks. Darker areas indicate higher probabilities of randomly finding a node with that degree and BiBC. Family assignment of the top ASVs in the network: ASV1, *Muribaculaceae*; ASV49, *Lachnospiraceae*. ASVs: Amplicon sequence variants; WD: Western diet; WD+Ch: WD with cholestyramine; BiBC: bipartite betweenness centrality.

Networks are commonly used in systems biology to identify relationships between hosts and the gut microbiota^[16,33,39]^. We implemented the Transkingdom Network Analysis (TkNA) pipeline^[13]^ to reconstruct a transkingdom network that included interactions (correlations) between the host parameters (both metabolic parameters and genes) and the gut microbiota of the cholestyramine-treated mice [Figure 4A]. The network consisted of 84 nodes and 154 edges after filtering out nodes and edges that did not satisfy statistical parameters (see methods) and causality principles (correlation inequalities^[29,30]^). The network was composed of 73 ASVs (53 of which were in the main component), 6 metabolic parameters, and 5 genes. The main component of the network included major parameters of glucose metabolism and 3 genes, 2 of which were from ileum. As expected for a biological network, the relationship between the number of nodes and the number of edges followed a power-law distribution [Supplementary Figure 5].

To determine which microbiota members were most likely to have a regulatory effect on the host response to cholestyramine, we focused on two key node properties: degree (the number of nodes a given node connects to) and BiBC. BiBC is similar to betweenness centrality, but BiBC splits the network up into groups (i.e., microbiota and metabolic parameters^[33,40]^). BiBC is essentially a measure of the “bottleneck-ness” of a node. The BiBC of a node counts how many times that node lies on the shortest paths between all possible pairs of nodes formed by selecting one node from the first group and one node from the second group. A microbe with a high BiBC and large degree is one that likely plays a role in host response to cholestyramine. By comparing BiBC and the degree of each parameter (using microbiota and host metabolic parameters as our two groups for the BiBC analysis), we found two potentially important ASVs [Figure 4A-D]. ASV49, which had both a large BiBC and degree, positively correlated with *Gcg* expression in the ileum and negatively correlated with both *Shp* in the liver and glucose 120 min in the glucose tolerance test. ASV1, which did not have a large BiBC but did have a high degree, primarily correlated with other ASVs.

We next used multiple methods to better define taxonomy for both ASV49 and ASV1 as described in Methods. The results of each method can be found in Supplementary Table 1. ASV1 was assigned to the *Muribaculaceae* family by four out of six methods. ASV49 was assigned to the *Lachnospiraceae* family by five of the six methods and to *Acetatifactor* genus by two of the methods. Both ASVs belonged to the first heatmap group [Figure 3] and their abundance was changed by Cholestyramine, with ASV49 increased and ASV1 decreased in the WD+Ch group from that of the WD group [Figure 4D].

To further validate our findings that ASV49 and ASV1 were important players in the host response to cholestyramine, we generated an ensemble of 10,000 random networks (see methods) and compared the BiBC and degree of the two microbes in the real network to the nodes with the largest BiBC and degree in the randomly generated networks [Figure 4E]. As predicted, the actual BiBC and degrees of ASV49 and ASV1 were atypical of those found in a random network (nodes with degrees and BiBC values that were high were extremely rare in the random ensemble). Meanwhile, those nodes that had a smaller BiBC and degree were clustered more toward the center of the BiBC-degree distribution, indicating they were not as important in the functional role of cholestyramine on the host.

On the gene expression part of the network, *Gcg* gene expression in the ileum and *Shp* gene expression in the liver were top candidates in regulating the host-microbiota interactions in response to cholestyramine [Figure 4B and E].

## DISCUSSION

From our study, it is clear that cholestyramine has more than just anti-hypercholesterolemic effects on the host. Consistent with findings from other studies, it can also help restore the ability of obese mice to metabolize glucose^[6,41]^. Furthermore, other bile acid sequestrants such as colesevelam, colestilan, and sevelamer have been shown to improve glucose levels in diseased individuals^[42-45]^. Consistent with our findings, it has previously been shown that cholestyramine can exert its anti-hyperglycemic effects via increasing ileal expression of *Gcg*, which encodes for preproglucagon, a precursor to GLP1^[46]^. Another consistent finding is that of an increase in alpha diversity of the gut microbiome following treatment with cholestyramine^[47]^. Our results of network analysis point to the possibility that changes in microbiota by cholestyramine, in particular the increase in ASV49, could stimulate *Gcg* gene expression, a hypothesis that can be tested in future work.

Given the limitations of 16S rRNA gene sequencing, we attempted to better understand the identity of our two top microbial candidates. BLAST identified ASV49 (*Lachnospiraceae* family) as most likely belonging to *Acetatifactor* genus, possibly *A. muris* species. Interestingly, *A. muris* was first identified in the caecum of obese mice and was noted to be a producer of both acetate and butyrate^[48]^. Previous research has demonstrated the importance of acetate in reducing fat accumulation in the liver and improving host response to insulin^[49]^. Similarly, butyrate administration in the diet corresponds with increased insulin sensitivity in mice^[50]^ and reduced cholesterol, low-density lipoprotein (LDL), very low-density lipoprotein (VLDL), and plasma triglycerides in diabetic rats^[51]^. Others have proposed that the main role of butyrate is to reduce the expression of genes involved in the synthesis of intestinal cholesterol^[52]^. This is in agreement with our observation of the increased relative abundance of *Acetatifactor* and decreased levels of cholesterol in cholestyramine-treated mice. Indeed, it seems the role of the *Acetatifactor* genus on the host may be multifaceted, as our study also corroborated previous findings that the genus positively correlated with fasting glucose levels. Furthermore, *Acetatifactor* is also known to be involved in bile acid transformations in the gut and was increased in mice treated for metabolic disease^[53-55]^. In addition, members of the *Lachnospiraceae* family, such as *A. muris*, can produce short-chain fatty acids (SCFA), and recently, it has been shown that treatment with cholestyramine in primary biliary cholangitis patients can increase the abundance of species in this family, potentially decreasing inflammation^[56]^. Notably, the same study also reported an increase in GLP-1 (GCG), consistent with what we observed. Interestingly, other studies have shown an opposite effect of *Lachnospiraceae*, in which *Lachnospiraceae* abundance increased in a high-fat diet and decreased in cholestyramine-treated mice on a matched high-fat diet^[57]^. This discordance indicates that there is likely species-level variability within the *Lachnospiraceae* family.

In contrast to ASV49, ASV1 was not consistently assigned beyond the family *Muribaculaceae*. The results of the transkingdom network indicate that while ASV1 may also play a role in crosstalk between microbiota in response to cholestyramine, because of its relatively low BiBC value and high degree, ASV1 does not appear to have a direct regulatory role on host metabolic parameters as ASV49.

**Figure 5:**
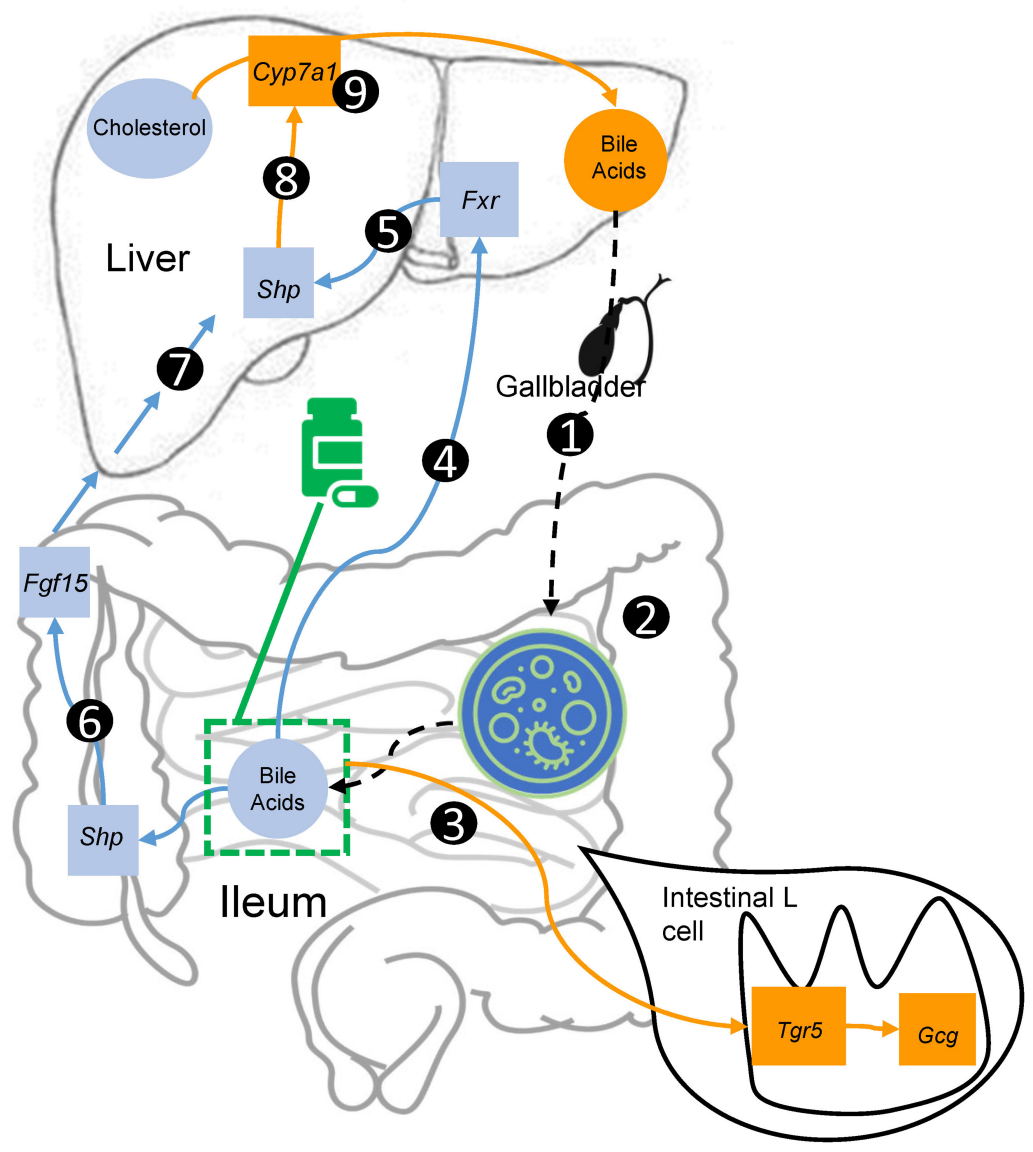
Proposed mechanism of how cholestyramine interacts with the gut microbiota, changing intestinal and hepatic gene expression to reduce cholesterol levels. (1) Bile acids from the liver, stored in the gallbladder, are secreted into the ileum following food consumption; (2) In the ileum, primary bile acids are transformed into secondary bile acids by microbiota; (3) Cholestyramine sequesters bile acids in the ileum where *Gcg* is expressed; (4) Enterohepatic circulation causes bile acids to be delivered from the ileum directly to the liver, where they bind to hepatic *Fxr* (*Nr1h4*); (5) Decreased *Fxr* expression in the liver causes decreased expression of Shp in the liver; (6) Decreased expression of Shp in the ileum and decreased expression of *Fgf15*; (7) This eventually leads to decreased expression of *Shp* in the liver; (8) Decreased *Shp* (a *Cyp7a1* inhibitor) in the liver causes increased expression of *Cyp7a1*; (9) *Cyp7a1* converts cholesterol in the liver to bile acids. *Gcg*: Glucagon; *Shp*: small heterodimer protein; *Fxr*: farnesoid X receptor; *Nr1h4*: nuclear receptor subfamily 1 group H member 4; *Fgf15*: fibroblast growth factor 15; *Cyp7a1*: cytochrome P450 family 7 subfamily A member 1.

The changes of gene expression associated with bile acid metabolism in the cholestyramine group observed are consistent with those one would expect to see with a bile acid sequestrant [Figure 5]. Specifically, the decreased availability of bile acids in the ileum (through sequestration by cholestyramine) decreases the activity of ileal *Fxr* (*Nr1h4*), which in turn would lead to decreased expression of ileal small heterodimer partner *Shp* (gene symbol *Nr0b2*^[58]^) and fibroblast growth factor 15 (*Fgf15*, the ortholog of *FGF19* in humans), as *Fxr* is a positive regulator of *Fgf15*. Lower expression of *Fgf15* in the ileum would lead to lower expression of *Shp* in the liver^[38,59]^. Since hepatic *Shp* normally inhibits *Cyp7a1*, *Cyp7a1* gene expression increases following the inhibition of *Shp* with cholestyramine^[60]^. Hence, a decreased expression of ileal *Fgf15* via cholestyramine administration leads to an increased expression of *Cyp7a1*.

Another non-mutually exclusive pathway for bile acid metabolism through *Cyp7a1* mediation has been proposed, in which enterohepatic circulation causes bile acids from the ileum to be transported to the liver, where they bind to activate *Fxr*, which then inhibits *Shp*^[61]^. The downstream target of *Fxr*, *Fgf15*, exhibited a decreased expression in the ileum, resulting in decreased *Shp* expression (both ileal and liver) and, thus, a higher expression of *Cyp7a1*. Interestingly, while we did not observe an increase in ileal *Tgr5*, its downstream target^[62]^, *Gcg* (GLP-1), was increased. It has also been hypothesized that bile acid sequestrants increase fatty acids in the ileum through decreased micelle production^[63]^. In turn, the increased availability of fatty acids may cause increases in *Gcg* expression^[64]^. Although we did not see significantly lower levels of *Fxr* expression in the ileum, it has been previously shown that colesevelam, another bile acid sequestrant, can inhibit L-cell *Fxr* expression, which in turn can act to increase *Gcg* expression^[65]^.

Our results are consistent with other studies showing that statins and bile acid sequestrants can modify the microbial composition of the gut^[4,66,67]^. Considering the crucial role of the gut microbiota in metabolizing bile acids (including deconjugation and hydrolysis^[68]^, the fact that fluctuations in bile acid sequestration levels lead to changes in the gut microbiota is not entirely surprising. It is well-known that bile acids are toxic to microbial cells, which requires the microbiota to chemically modify the bile acids as a defense mechanism^[69]^. By binding to bile acids, cholestyramine and other bile acid sequestrants may decrease selective pressure on host microbiota, resulting in the increase in alpha diversity we observed in the WD+Ch group.

Typical for an exploratory study, this work also has limitations that constrain the strength of conclusions drawn from the analyses. First, we report the results of a meta-analysis of two experiments with a small difference in the study timeline (19 *vs*. 18 total weeks) due to unforeseen personnel reasons. This difference may cause some false-negative results (decrease the number of significant differences) but would not generate false positive results. Furthermore, we observed that most of the metabolic and gene expression changes between the groups had the same direction and similar values across the two experiments [Figure 1]. Therefore, while this limitation reduced the power of analysis, it simultaneously increased the generalizability of our findings.

The second drawback is the lack of validation of the causal inferences regarding the predicted effects of candidate microbes (such as ASV49) involving bacterial administrations. Another limitation is related to the use of 16S rRNA gene sequencing that can give limited and/or outdated taxonomic assignments in some cases. One example is the family S24*-*7, which was recently described as *Muribaculaceae*^[70]^, yet some methods have not changed the annotations, as can be seen in Supplementary Table 3. Finally, we had to use moderate strength of statistical thresholds for network reconstruction because of a limited sample size. This weakness, however, is counterbalanced by several factors. First, the standard approach (Benjamini-Hochberg FDR) used in our paper to control error rate due to multiple hypotheses is over-conservative for covariation networks as edges are not independent hypotheses. Accordingly, implementing Benjamini-Hochberg FDR for such cases always leads to an unjustified rise in false negative error rates. Second, the most critical inferences from network analysis (i.e., identification of hubs/nodes presenting high ranking degree and bipartite betweenness centrality) are typically quite resilient to the choice of specific statistical thresholds and do not depend much on the certainty of an individual edge in a network. Finally, using random network simulations, we evaluated whether highly ranked nodes in our network represented a robust finding and observed that the two key identified microbes could hardly be found due to random chance.

In conclusion, we have demonstrated that treatment of obese diabetic mice with the bile acid sequestrant, cholestyramine, improves systemic glucose metabolism, possibly through changes in the host microbiota. Mice treated with cholestyramine demonstrated significant changes in microbial composition as well as changes in the expression of genes involved in the bile acid metabolism pathway. Through reconstruction and interrogation of a data-driven transkingdom network, we were able to identify two potential ASVs responsible for the host response to cholestyramine. The next step is to perform experimental validation of these findings via the colonization of germ-free mice with each of the identified species to determine whether their presence is truly indicative of an individual’s response to cholestyramine. Our work has provided a foundation of knowledge for host-microbiota interactions under cholestyramine and can help in understanding the complex relationship between glucose, bile acid metabolism, and the gut microbiota.

## DECLARATIONS

### Availability of data and materials

16S sequencing data for both experiments were uploaded to SRA (Sequence Read Archive) and can be accessed through the BioProject accession number PRJNA686421 (https://www.ncbi.nlm.nih.gov/bioproject/PRJNA686421).

### Copyright

© The Author(s) 2024.
