# Supplementary material for: Host response to cholestyramine can be mediated by the gut microbiota
Source: Microbiome Res Rep. 2024 Jul 11;3(4):40. doi: 10.20517/mrr.2023.82 (PMC11684918; doi:10.20517/mrr.2023.82)
Supplement: Supplementary file 1 [file mrr-3-4-40-SupplementaryMaterials.pdf]

Supplementary Figures

Host response to cholestyramine can be mediated by the gut microbiota

Nolan K. Newman[1], Philip M. Monnier[1], Richard R. Rodrigues[1], Manoj Gurung[2], Stephany Vasquez-Perez[2], Kaito A. Hioki[1], Renee L. Greer[2], Kevin Brown[1], Andrey Morgun[1], Natalia Shulzhenko[2]

[1]Department of Pharmaceutical Sciences, College of Pharmacy, Oregon State University, Corvallis, OR 97331, USA.
[2]Department of Biomedical Sciences, Carson College of Veterinary Medicine, Oregon State University, Corvallis, OR 97331, USA.

Correspondence to: Dr. Natalia Shulzhenko, Department of Biomedical Sciences, Carson College of Veterinary Medicine, Oregon State University, 208 Dryden Hall, Corvallis, OR 97331, USA. E-mail: natalia.shulzhenko@oregonstate.edu

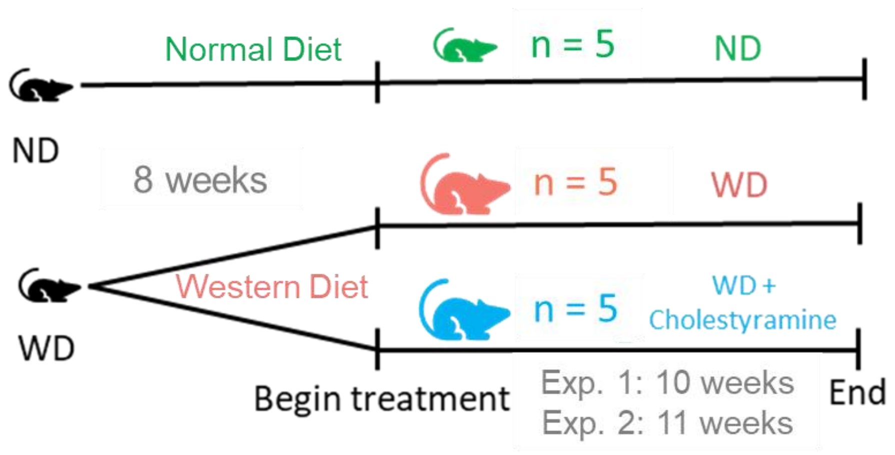

Supplemental Figure 1. Experimental design of the study that consisted of 2 experiments with 15 mice in each and total of 30 mice. In experiment 2, mice were treated with cholestyramine for 11 instead of 10 weeks because of the personnel health issues. ND, normal diet; WD, western diet.

Gene Name	Forward Primer (5'→3')	Reverse Primer (5'→3')
Fgf15	GCCATCAAGGACGTCAGCA	CTTCCTCCGAGTAGCGAATCAG
Gcg	TTACTTTGTGGCTGGATTGCTT	AGTGGCGTTTGTCTTCATTCA
Shp	TGGGTCCCAAGGAGTATGC	GCTCCAAGACTTCACACAGTG
Fxr (Nr1h4)	TGGGCTCCGAATCCTCTTAGA	TGGTCCTCAAATAAGATCCTTGG
Cyp7a1	TGGGGGATTGCTGTGGTAGT	AGCACAGCCCAGGTATGGAA
Tgr5	CTGTGTGAGATCCGCCGAC	CGACGCTCATAGGCCAAGA
Polr2c	CTCACCGAAGAGAACGTCAAG	TCGATGGCTATTATGGGCACC

Supplementary Figure 2. Primers used for quantitative RT-PCR and were designed by the lab.

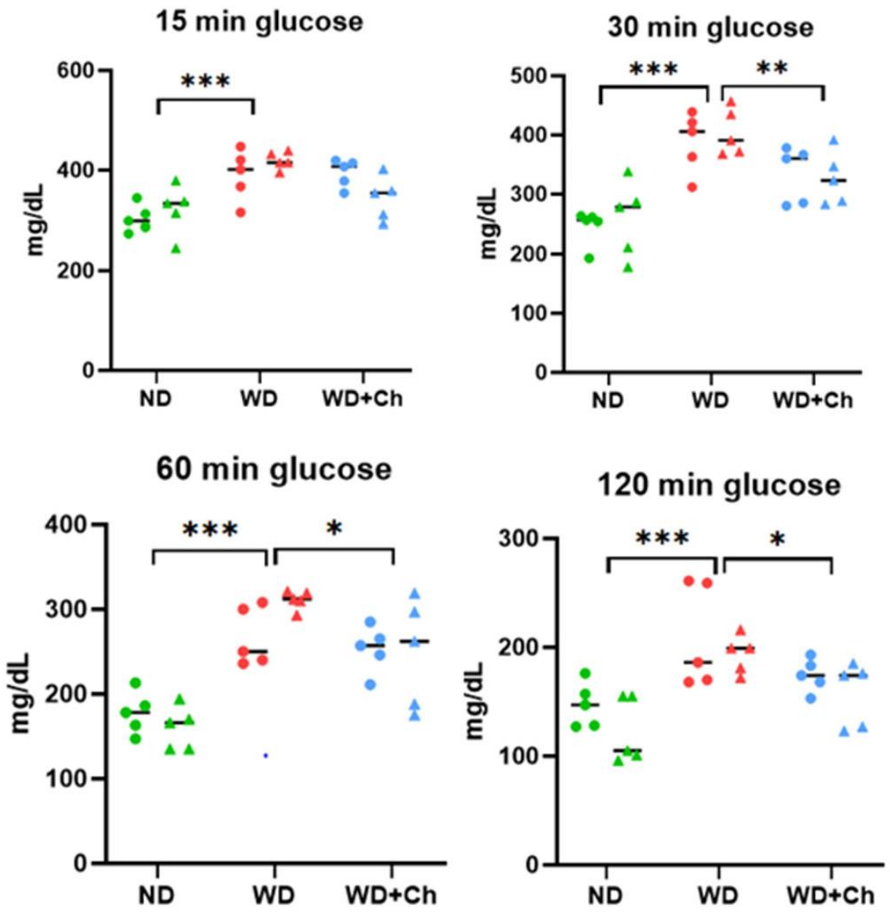

Supplementary Figure 3. Blood glucose levels 15, 30, 60, and 120 minutes after intraperitoneal glucose injection. Different symbol shapes indicate two experiments. ND, normal diet; WD, western diet; Ch, cholestyramine

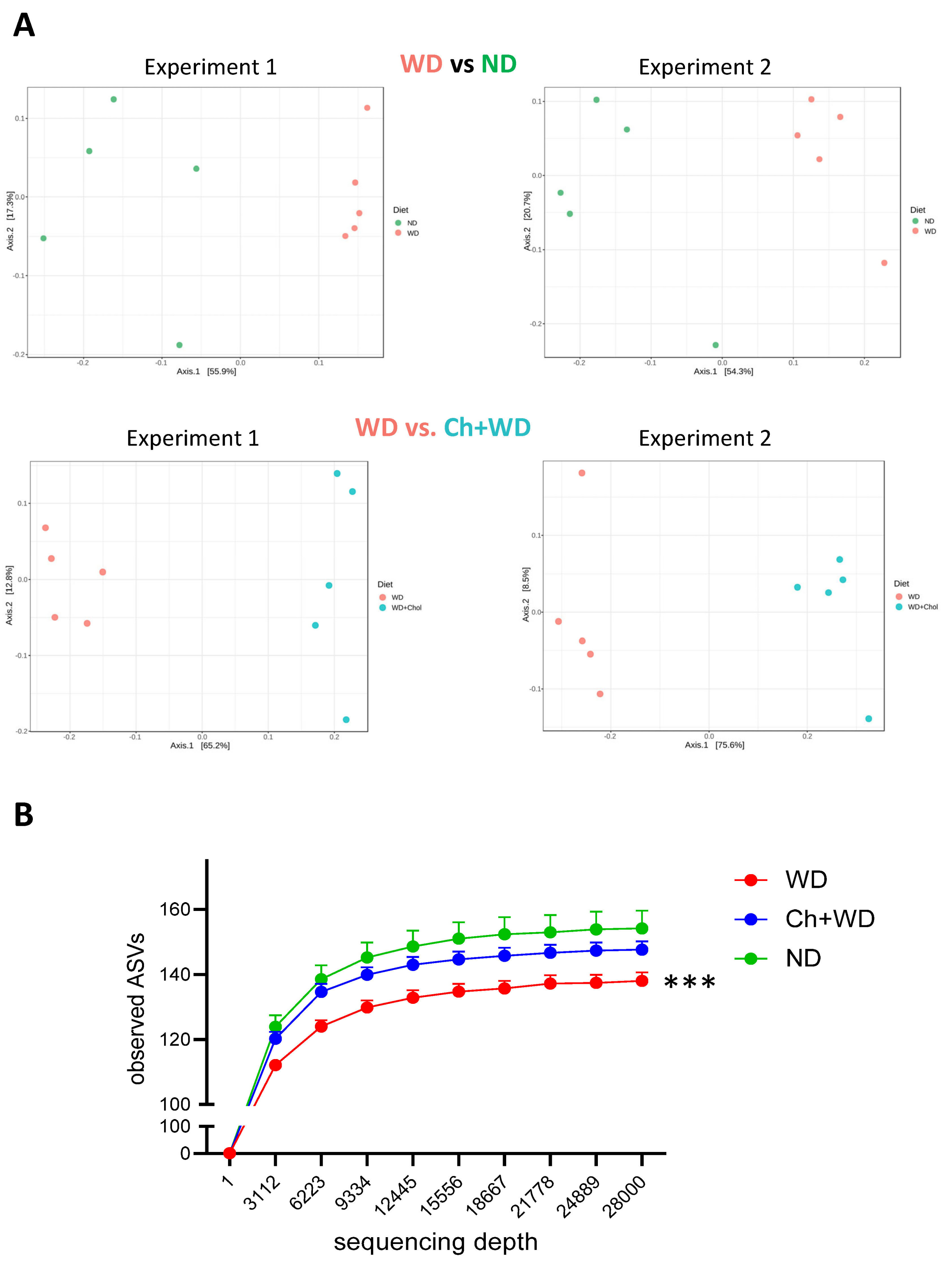

Supplementary Figure 4. (A) PCoA plots showing feature-level differences of ASVs between treatment groups; (B) Depth of sequencing and number of observed ASVs showing that WD group has lower richness than the other two groups. ***$P < 0.001$. ND: Normal diet; WD: western diet; Ch: cholestyramine.

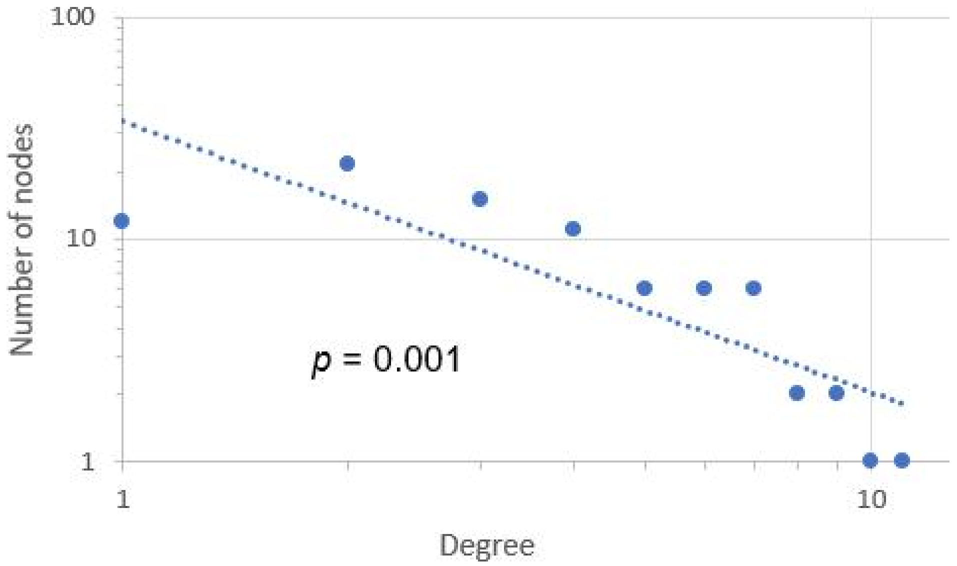

Supplementary Figure 5. The cholestyramine network follows a power-law degree distribution typical for a regulatory network.
